# Direct observation of isolated Damon-Eshbach and backward volume spin-wave packets in ferromagnetic microstripes

**DOI:** 10.1038/srep22117

**Published:** 2016-02-24

**Authors:** Philipp Wessels, Andreas Vogel, Jan-Niklas Tödt, Marek Wieland, Guido Meier, Markus Drescher

**Affiliations:** 1 The Hamburg Centre for Ultrafast Imaging (CUI), Luruper Chaussee 149, 22761 Hamburg, Germany; 2 Center for Optical Quantum Technologies (ZOQ), University of Hamburg, Luruper Chaussee 149, 22761 Hamburg, Germany; 3 Institut für Nanostruktur- und Festkörperphysik (INF), University of Hamburg, Jungiusstraße 11, 20355 Hamburg, Germany; 4 Institut für Experimentalphysik, University of Hamburg, Luruper Chaussee 149, 22761 Hamburg, Germany; 5 Max-Planck-Institute for the Structure and Dynamics of Matter, Luruper Chaussee 149, 22761 Hamburg, Germany

## Abstract

The analysis of isolated spin-wave packets is crucial for the understanding of magnetic transport phenomena and is particularly interesting for applications in spintronic and magnonic devices, where isolated spin-wave packets implement an information processing scheme with negligible residual heat loss. We have captured microscale magnetization dynamics of single spin-wave packets in metallic ferromagnets in space and time. Using an optically driven high-current picosecond pulse source in combination with time-resolved scanning Kerr microscopy probed by femtosecond laser pulses, we demonstrate phase-sensitive real-space observation of spin-wave packets in confined permalloy (Ni_80_Fe_20_) microstripes. Impulsive excitation permits extraction of the dynamical parameters, i.e. phase- and group velocities, frequencies and wave vectors. In addition to well-established Damon-Eshbach modes our study reveals waves with counterpropagating group- and phase-velocities. Such unusual spin-wave motion is expected for backward volume modes where the phase fronts approach the excitation volume rather than emerging out of it due to the negative slope of the dispersion relation. These modes are difficult to excite and observe directly but feature analogies to negative refractive index materials, thus enabling model studies of wave propagation inside metamaterials.

Today’s advanced electronic devices operate at high frequencies to complete complex tasks on reasonable time scales. These circuits solely use the charge of the electron to transport and process information. Future computer and memory concepts may use the electron spin instead of or in addition to its charge for data transport and storage as well as logical operations^1^,^2^,^3^,^4^,^5^. Such spintronic and magnonic devices benefit from the fact that frictional heating in magnetic transport is negligible compared to charge transport in conventional electronic devices where the resulting heat load imposes serious limitations in building ever smaller and faster circuits. Spin-waves appear as an ideal solution for fast magnetic information transport since their natural frequencies in the gigahertz regime are compatible with clock rates of modern computer processors and mobile communication technology. Extensive research has been carried out both theoretically and experimentally to characterize the dynamic parameters of spin-waves and first logic devices have been realized^3^,^6^,^7^,^8^,^9^,^10^,^11^,^12^,^13^ in which the spin-wave is excited locally via an Oersted field^14^,^15^,^16^ or through spin-torque^17^,^18^,^19^. Many studies consider the steady state where a spin-wave is continuously excited at microwave frequencies. Information transport and processing, however, relies on the dynamical modulation of a wave’s amplitude or phase. For the realization of applicable spintronic devices, knowledge about the transient features is mandatory, i.e. spin-wave packets confined in space and in time are to be considered^11^,^20^,^21^,^22^,^23^.

In addition to fundamental insight into magnetic transport, spin-wave packets can serve as a simulator toolkit for electromagnetism where tunable dispersion relations in confined structures^24^,^25^,^26^ give rise to unusual wave packet dynamics. Backward volume (BV) spin-wave packets with small wave vectors 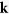, for example, exhibit a phase front motion with a phase velocity 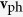 opposite to the propagation direction associated with the group velocity 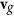. A negative scalar product in form of 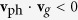 is equivalent to a negative refractive index 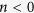 in the optical regime^27^,^28^,^29^. This conceptual similarity shall enable model studies of waves^30^ also in the field of metamaterials where negative phase velocities occur^31^,^32^.

Parameters of spin excitations like spatial dimensions and damping mainly depend on the choice of materials and excitation mechanisms. Spin-waves in monocrystalline ferrimagnetic yttrium iron garnet 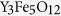 (YIG) films^33^ can be efficiently excited and feature a low damping constant; however, manufacturing of the monocrystalline layer is demanding and the wavelength is typically in the mm-range. Metallic ferromagnets like permalloy (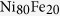) support micro-21,22,34,35 and nanoscale^17^,^18^,^19^ excitations meeting the size of functional units in computer and communication devices dictated by the high quantity of embedded logical units. The majority of experimental studies in ferromagnetic thin films focuses on Damon-Eshbach (DE) spin-waves^36^ where an external magnetic bias field 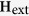 is applied perpendicular to the propagation direction. Although backward volume (BV) spin-waves are most efficiently supported with a bias field parallel to the propagation path, they may even propagate without an external field^37^ and are therefore desirable candidates for logic devices. BV spin-wave modes have been reported in YIG^38^ including the observation of wave-packets^39^,^40^ and solitons^41^,^42^,^43^,^44^. Downscaling of spin-wave dynamics using metallic ferromagnets is possible but the generation and control of BV wave packets is difficult^45^,^46^,^47^,^48^ due to the lower excitation efficiency^37^ and the small group velocity. Thus, a direct spatiotemporal observation of BV modes with counterpropagating phase- and group-velocity remains challenging and is often only indirectly achieved out of propagating DE spin-waves^49^,^50^,^51^. Recently, BV modes with counterpropagating phase- and group-velocities have been observed in ferrimagnetic insulators via direct excitation through circularly polarized femtosecond laser pulses^52^. Our work reports on similar excitations in metallic ferromagnets through a dedicated excitation scheme enabling the propagation of dispersive BV spin-wave packets with considerably smaller wavelength and damping. This is achieved through a novel optically driven high-current pulse source supporting excitations up to a bandwidth of 50 GHz via short magnetic field pulses of <70 ps FWHM duration, perfectly synchronized to the sub-ps optical probe pulses.

## Results

### Experimental setup

**Figure 1:**
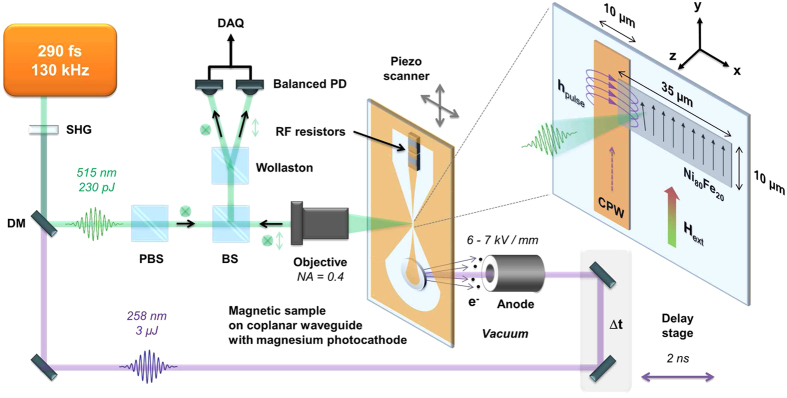
Schematic of the time-resolved scanning Kerr microscope (TR-SKM) and the sample. Visible pulses of a highly repetitive femtosecond laser amplifier are converted into the UV spectral range via second harmonic generation (SHG) to trigger a photocathode using the photo effect and an electrostatic extraction field. This allows the generation of intense and broadband current pulses for spin-wave excitation through a coplanar waveguide (CPW) terminated in two radio-frequency (RF) resistors. The residual visible light pulses are separated by a dichroic mirror (DM), polarization filtered by a polarizing beam splitter (PBS), and focused onto the magnetic sample layer. The reflected light is recollected and directed into a Wollaston prism through an intensity beam splitter (BS). The polarization change due to the magneto-optic Kerr effect (MOKE) is analyzed and collected by the data acquisition (DAQ) in a balanced photodetector (PD). The sample can be scanned through the probe laser focus by a two-axis piezo scanner.

Combining magnetic microscopy with an excite & probe technique allows us to directly observe the spatiotemporal evolution of isolated wave packets. The experimental setup shown in Fig. 1 comprises a time-resolved scanning Kerr microscope^53^ (TR-SKM) detecting the transient magnetization response normal to the surface (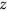-direction) through the magneto-optic Kerr effect (MOKE)^54^,^55^, probed with femtosecond laser pulses in the visible spectral range (515 nm wavelength). In this work we excite spin-waves in an impulsive manner through a novel optically driven current source^53^. The green laser pulses are doubled in frequency to create synchronized, delayable ultraviolet (UV) pulses sufficiently energetic (4.8 eV photon energy) for releasing photoelectrons from a magnesium (Mg) surface exposed to a static electric field in a high-vacuum environment. The Mg photocathode is attached to a tapered coplanar waveguide (CPW), transporting the intense broadband current pulses to a magnetic sample in form of a rectangular permalloy layer of 35 × 10 µm^2^ dimension and 30 nm thickness in 5 μm partial overlap with the waveguide conductor (see magnified inset in Fig. 1). These pulses are terminated in a serial connection of two radio-frequency resistors with 50 GHz bandwidth. Since current source, termination, magnetic sample and waveguide are combined on a single sapphire substrate, the choice of the waveguide impedance is arbitrary and enables the preparation and investigation of complex sample geometries. Here, an overall impedance of 100 Ω was chosen resulting in a CPW with a central 10 μm wide conductor surrounded by two ground planes separated by a gap of 48.5 μm, large enough to fit the permalloy sample layer without shorting conductor and ground striplines (see Supplementary Fig. S1 for details of the central waveguide part). This excitation approach differs from established photoconductive switches such as an Auston switch commonly used for triggering spin precession dynamics^21^,^56^. The photocathode achieves superior peak currents^53^ exceeding 0.5 A and avoids asymmetric pulse shapes as found in Auston switches due to long carrier lifetimes. Such pulses may influence the magnetization dynamics by a residual dynamic bias field in the trailing edge of the pulse^21^,^56^ (compare also Supplementary Fig. S4). However, the photocathode can only be operated in an ultra-high vacuum environment and requires laser pulses in the UV spectral range.

**Figure 2:**
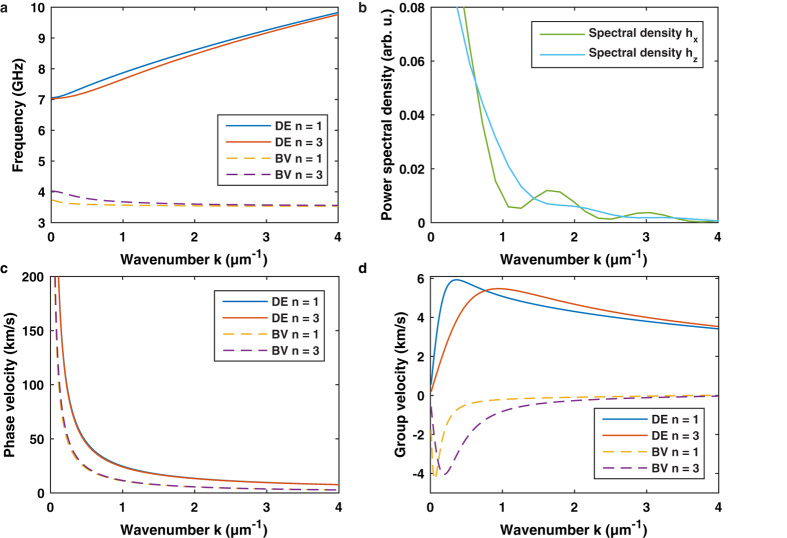
Spin-wave dispersion relation with excitation spectrum and derived phase- and group velocities. (**a**) Dispersion relation 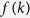 for the first two quantized transversal Damon-Eshbach (DE) (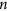 = 1 blue solid line, 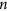 = 3 orange solid line) and backward volume (BV) (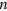 = 1 yellow dashed line, 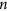 = 3 purple dashed line) spin-wave modes of a permalloy (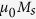 = 1.04 T, 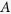 = 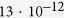 J/m) stripe with an effective width of 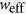 = 10.12 μm, an effective length of 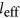 = 35.1 μm and 30 nm thickness. The DE modes are calculated for an external field of 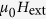 = 60 mT amplitude reduced by the internal demagnetization field while the BV modes are calculated for an external field of 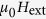 = 16 mT amplitude. (**b**) Power spectral density 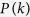 of the excited wavenumbers 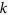 by the magnetic field pulse determined by the Fourier transformation of the spatial magnetic field configuration for the 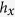 component (green solid line) and the 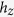 component (cyan solid line). (**c**) Phase velocities 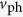 = 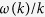 for all calculated spin-wave modes. (**d**) Group velocity 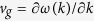 for all calculated spin-wave modes.

The sample magnetization is prealigned by an external bias field 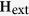 generated by a set of permanent magnets and locally modified by the dynamic field of the current pulse 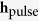 in the overlap region of waveguide conductor and permalloy layer. Once exposed to the excitation field, the magnetic moments start to precess; due to their mutual interaction this precessional motion is transferred to neighboring magnetic moments thus releasing a spin-wave packet. The response of the spin system is governed by the frequency *f* and the spatial field configuration of the source. While spatial dimensions dictate the excited wavelengths *λ* or wavenumbers 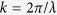, the angular frequency 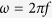 drives the gyration motion of the magnetic moments. Only certain combinations of frequency and wave vector 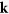 allowed by the dispersion relation 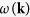 plotted in Fig. 2a may lead to a propagating dispersive spin-wave packet. Here, the impulsive magnetic excitation emerges from femtosecond laser pulses and the pulse duration is limited by the bandwidth of waveguide and termination resistors as well as space charge effects in the photocathode and excitation pulses of 70 ps FWHM duration are achievable leading to a broadband excitation spectrum (see Methods section). Thus, the propagating spin-wave is dominated by the geometry of the excitation field given by its Fourier transform^21^,^22^ shown in Fig. 2b. A spin-wave packet is then composed of the superposition of all excited wave vectors 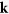 convoluted by the excitation probability in this spectrum 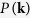. The observable *z* component 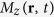 reads


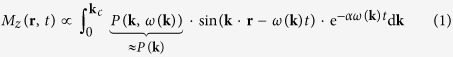


with a damping term^52^ related to the dimensionless damping parameter *α* = 0.008 for permalloy shown in equation (1). A cut-off wave vector 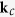 can be introduced for numerical integration because the excitation probability approaches zero for high wave vectors. Due to the broad frequency spectrum, the dispersion relation 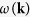 defines the gyration frequency *f* connected to the specific wave vector 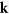.

In addition to DE modes with co-propagating phase- and group velocities, high peak amplitudes enable us to also observe BV modes where the phase fronts do not emerge out of, but rather approach the excitation volume. This unusual fact emerges from the negative slope of the BV dispersion relation in Fig. 2a formally related to a negative group velocity 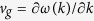 counteraligned to the phase front motion. Before focusing on the backward volume spin-wave packets, we demonstrate the capabilities of our setup by mapping the evolution of Damon-Eshbach modes.

### Damon-Eshbach spin-wave packets

**Figure 3:**
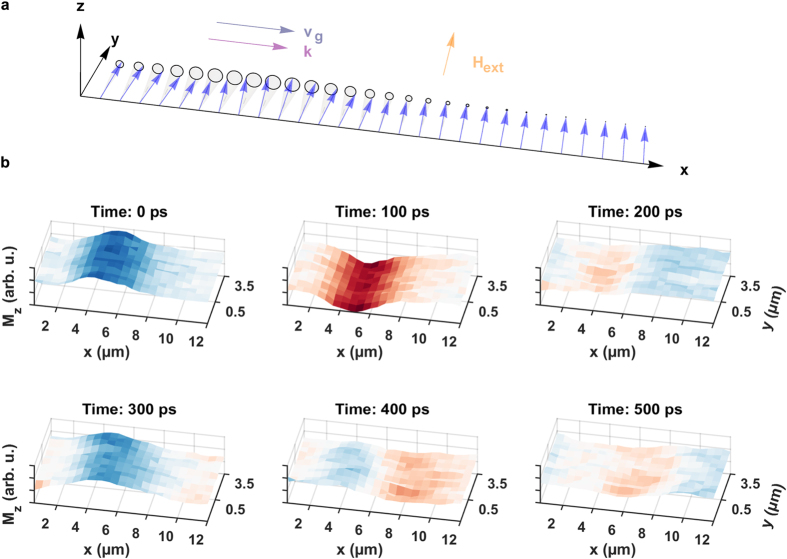
Propagation of a Damon-Eshbach spin-wave packet. (**a**) Precessional motion of the magnetic moments in a Damon-Eshbach spin-wave packet. The propagation direction of the phase fronts is determined by the wave vector 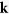 which is perpendicular to the external bias field 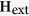 and parallel to the group velocity 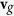 determining the propagation direction of the wave packet. (**b**) Selected frames of the Damon-Eshbach spin-wave movie illustrating the real space time evolution of the out-of-plane magnetization component 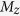 at six delay settings. In contrast to the frames in Fig. 6b the phase fronts emerge out of the excitation region toward higher 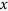 coordinates (see also Supplementary Video S5).

Figure 3 presents an illustration of the precessional motion (a) together with selected frames of a captured DE spin-wave packet (b) of Supplementary Video S5. Here, the external bias field is oriented in *y* direction, perpendicular to the propagation direction of the wave packet. The width of the permalloy layer extends from *y* = −5 to 5 μm with the center located at *y* = 0 μm. The overlap with the waveguide conductor alongside the *y* axis ranges from *x* = 0 to 5 μm. Upon arrival of the dynamic field pulse at the time *t* = 0 ps, the 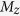 component of the magnetization starts to oscillate in a spatially confined source region where the permalloy structure partially covers the waveguide. As time elapses, a wave packet is released and propagates down the *x* axis.

**Figure 4:**
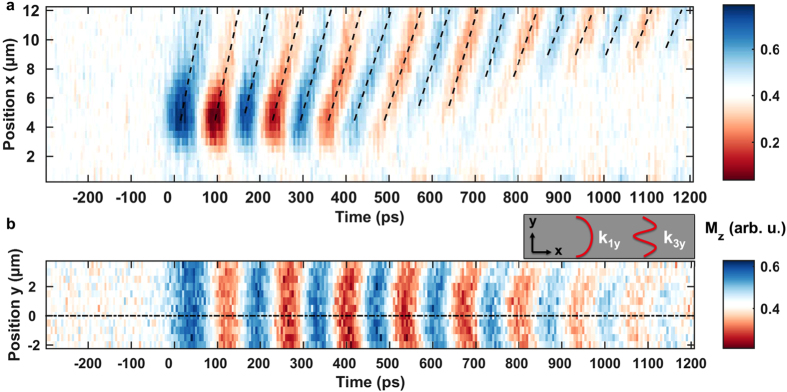
Details of the DE spin-wave packet propagation in a permalloy stripe. The color code identifies the detectable transient 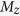 component of the magnetization and the static bias field of *μ*_0_*H*_ext_ ≈ 60 mT amplitude is aligned in 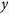 direction. (**a**) Propagation in 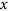 direction at a fixed transverse position 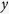 = 0 μm in the center of the slab with respect to time. Black dashed lines trace the maxima and minima of the wave and thus relate to the phase velocity 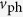. (**b**) Temporal evolution of the spatial transverse mode distribution at a fixed longitudinal position (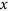 = 8 μm). The amplitude of the first two modes 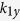 and 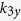 is depicted in the inset. The center of the slab marked by the black dash-dotted line is located at 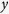 = 0 μm slightly off from the center of the 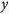 scan range in this measurement.

This process is depicted in more detail in Fig. 4. Here, the color coded detectable 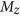 component is plotted with respect to time and one spatial coordinate. Figure 4a shows the spin-wave propagation 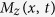 in *x* direction along a fraction of the extended 35 μm long permalloy slab axis at a fixed transverse coordinate *y *= 0 μm. With evolving time, new phase fronts emerge out of the excitation region (0 to 5 μm) toward larger *x* coordinates leading to a positive slope in the 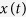 diagram in Fig. 4a when tracing the oscillation maxima and minima. An evaluation of the slopes in terms of straight lines (black dashed lines in Fig. 4a) yields phase velocities ranging from 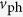 = 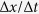 = 70 to 150 km/s (see Supplementary Fig. S2a). With elapsed time, the oscillation amplitude decreases in the source region around *x* = 5 μm and almost vanishes there after 600 ps. But due to the interaction of the magnetic moments, a spin-wave packet starts to propagate toward increasing *x* positions. However, the oscillation there is not as confined in time as in the source region because the wave packet spreads. Additionally, the plot enables an extraction of the 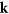-vector magnitude by measuring the distance 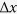 between an oscillation maximum and minimum which is related to half the wavelength 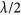 and thus to the wave vector magnitude via *k* = 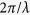. An evaluation yields *k* = 0.3 to 0.6 μm^−1^ in accordance with the excitation spectrum due to the waveguide overlap with the permalloy layer.

Figure 4b traces the oscillation 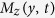 at a fixed longitudinal position of *x* = 8 μm and shows the temporal evolution of the spatial modes along the 10 μm width of the permalloy structure parallel to the *y* axis. We observe a transition from an initially constant phase over the slab into a curved phase front. This self-focusing of the spin-wave packet can be explained by an interference of the dominant first two quantized transverse modes^34^,^35^,^57^ with a wavenumber of 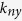 = 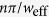 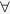 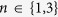 since only odd modes can be excited by a uniform radio-frequency field^58^. The effective stripe width 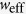 is introduced to account for dipolar pinning effects at the sample boundaries reducing the magnetization because of the demagnetization field^25^ and here computes to 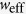 = 10.12 μm. Knowing that the amplitude of the mode scales with^58^ 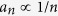, we can model the response of the magnetization oscillating with a frequency *ω* by^35^,^57^


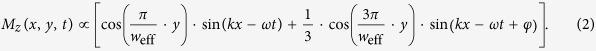


The first two transversal modes corresponding to the two cosine terms in equation (2) are depicted in the inset of Fig. 4b. In general, these modes will be phase-shifted by 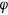 with respect to each other. In this model, a phase shift of 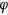 = 40° reproduces the observed curvature in the experiment.

**Figure 5:**
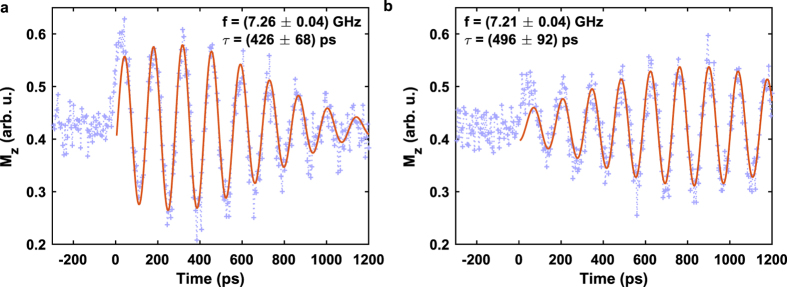
DE spin-wave packets. (**a**) At position 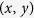 = (8 μm, 0 μm) closer to the source region, and (**b**) at position (12 μm, 0 μm) further away from the source region in the center of the slab. The blue crosses represent the measured Kerr signal and the red solid line shows a fit to the data according to equation (3). The peak amplitude of the wave packet in (**b**) is reduced to 70% of the wave packet amplitude in (**a**).

Plotting the 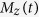 component with respect to time at two different points 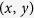 = (8 μm, 0 μm) and (12 μm, 0 μm) in the center of the slab along the propagation direction of the wave packet in Fig. 4a helps to further clarify the evolution of the wave packet. The corresponding TR-SKM data is shown in Fig. 5 including a fit according to


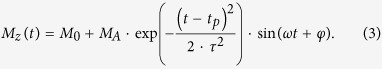



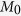
 is an offset of the magnetization signal, 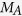 determines the peak amplitude of the wave packet, 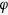 a phase offset, 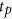 the time of the wave packet envelope maximum, *τ* the root mean square (rms) wave packet duration and *ω* = 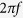 the central angular oscillation frequency. Data points at negative time delays before the arrival of the excitation pulse have been excluded from the fit.

This method enables a global transient analysis of the above mentioned spin-wave parameters during formation and propagation of the wave packet. In particular, we are able to extract the carrier frequency *f* ≈ 7.2 GHz which is in good agreement with the spin-wave dispersion relation in Fig. 2a and it is obvious that the wave packet duration *τ* increases while the peak amplitude 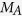 decreases along its way down the *x* axis. Furthermore, the center of the wave packet envelope 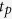 shifts to a later instance in time for an increased distance *x* from the source region meaning that it takes more time to travel to a distance further away from the excitation field. Hence, the group velocity vector points into the same direction as the phase velocity vector, namely into positive *x* direction. By relating the center of the wave packet 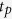 to the propagated distance *x* we can extract the conveyor speed of the whole wave packet which is described by the group velocity


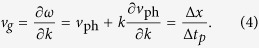


Applying the fit of equation (3) to the measured wave packet data at different distances *x* provides a linear relation between *x* and 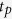 (see Supplementary Fig. S2b) and yields a positive group velocity of 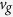 = (6.0 ± 0.8) km/s. This velocity is consistent with the group velocity calculated by computing the derivative of the spin-wave dispersion relation in Fig. 2d for the observed wavenumbers.

With knowledge of the spin-wave packet’s oscillation frequency, the phase velocity can also be calculated via


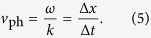


By inserting the extracted wavenumbers from Fig. 4a (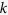 = 0.3 to 0.6 μm^−1^) and the carrier frequency (*f* ≈* *7.2 GHz), phase velocities ranging from 75 to 151 km/s are obtained in agreement with the directly extracted velocities of the phase fronts in Supplementary Fig. S2a and with the phase velocities calculated using the dispersion relation in Fig. 2c resulting in values between 70 and 150 km/s for wavenumbers between 0.2 and 0.4 μm^−1^.

### Backward volume spin-wave packets

**Figure 6:**
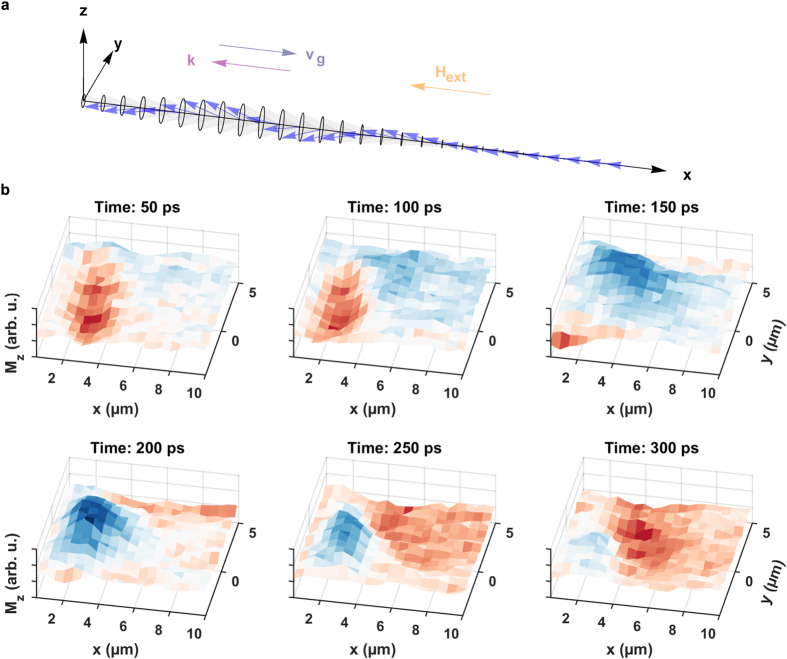
Propagation of a backward volume spin-wave packet. (**a**) Precessional motion of the magnetic moments in a backward volume spin-wave packet. The propagation direction of the phase fronts is determined by the wave vector 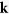 which is parallel to the external bias field 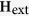 and antiparallel to the group velocity 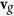 determining the propagation direction of the wave packet. (**b**) Selected frames of the backward volume spin-wave movie illustrating the real space time evolution of the out-of-plane magnetization component 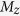 at six delay settings. It is evident that the phase fronts do not emerge out of the excitation region but rather approach the source from higher 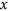 coordinates indicating a negative phase velocity (see also Supplementary Video S6).

For the acquisition of backward volume spin-wave packets, the external bias field is tilted by 90° and is now oriented in −*x* direction, antiparallel to the propagation direction of the wave packet. Note that the amplitude of the external field also changes due to the sample geometry (see Methods section). Figure 6 presents a space-time illustration of the precessional motion (a) together with selected frames of a captured BV spin-wave packet (b) of Supplementary Video S6. Upon arrival of the dynamic field pulse an oscillation of the 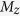 component is visible in the source region. As time elapses, a new phase front (in this case a blue color coded maximum) starts to approach the excitation region and propagates from larger to lower *x* coordinates. When the maximum has arrived, a minimum (red color code) enters the scan window from the right border heading to the overlap region. From the six frames in Fig. 6b it is already obvious that the phase velocity describing the motion of the oscillation maxima and minima is directed along the negative *x* axis.

**Figure 7:**
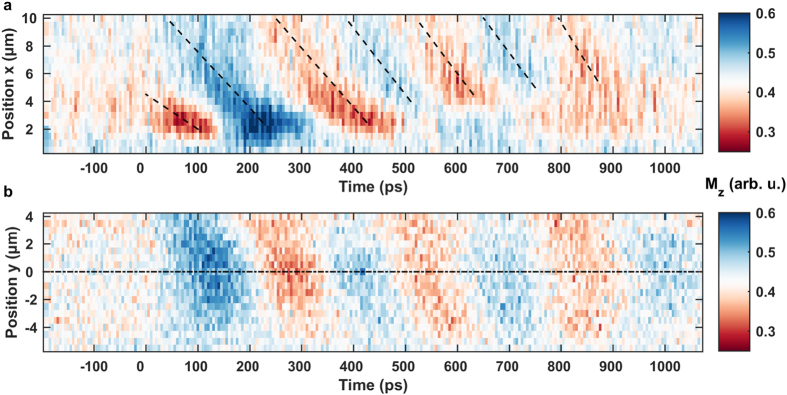
Details of the BV spin-wave packet propagation in a permalloy slab. The color code identifies the detectable transient 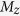 component of the magnetization and the static bias field of *μ*_0_*H*_ext_ ≈ 16 mT amplitude is aligned in 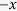 direction. (**a**) Propagation along the elongated 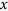 direction at a fixed transverse position 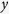 = 0 μm in the center of the element. Black dashed lines trace the maxima and minima of the wave and thus relate to the phase velocity 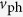. The negative slope of the phase fronts clearly substantiates a negative phase velocity. (**b**) Transverse spatial mode distribution at a fixed longitudinal position 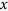 = 7 μm with respect to time. The black dash-dotted line at 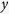 = 0 μm marks the center of the slab.

Figure 7 shows the BV spin-wave packet in the same representation used in Fig. 4 for the DE data. An oscillation of the magnetization can be observed in the overlap region of permalloy and waveguide conductor when the dynamic field pulse arrives at *t* = 0 ps and in this case the maxima and minima start to approach the excitation region from larger *x* coordinates with a negative phase velocity indicated by the slope of the black dashed lines. An evaluation of the slopes yields phase velocities ranging from 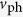 = 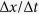 = −25 to −60 km/s (see Supplementary Fig. S3). The excited wave vector magnitude covered here is *k* = 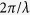 = 0.2 to 0.7 μm^−1^ corresponding to phase velocities ranging from 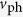 = 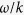 = −31 to −110 km/s (*f* ≈ 3.5 GHz) in agreement with the directly extracted slopes and with the derived phase velocities from the spin-wave dispersion relation in Fig. 2c between −25 and −60 km/s for wavenumbers between 0.2 and 0.5 μm^−1^.

For the spatial mode distribution 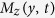 in Fig. 7b we observe an almost uniform shape and a decay of the oscillation amplitude with time. It is conspicuous that the phase fronts in this image are slightly tilted and that also the phase fronts in Fig. 6b enter the scan window from the top right corner. This means that the propagation direction is not completely parallel to the *x* axis as would be expected for a perfectly collinear alignment of the external field. An explanation is given by the sensitivity of the group and phase velocity direction on the external field alignment due to the shape of the isofrequency line to which excitable modes are restricted^24^,^59^. The observed group velocity vector tilt of approximately 10° corresponds to a magnetic field tilt of 8°. Furthermore, a perfect collinear alignment would result in a vanishing torque on the dipole moments from the 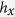 component of the dynamic field. The excitation would then be mainly driven by the 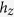 component.

**Figure 8:**
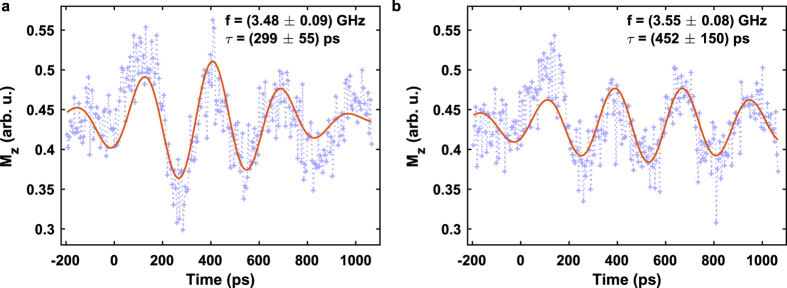
BV spin-wave packets. (**a**) At position 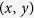 = (7 μm, 1 μm) closer to the source region, and (**b**) at position (10 μm, 1.5 μm) further away from the source region. The blue crosses represent the measured Kerr signal and the red solid line shows a fit to the data according to equation (3).

Similar to the Damon-Eshbach wave packets we can plot the 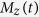 component with respect to time at two different points 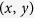 = (7 μm, 1 μm) and (10 μm, 1.5 μm) along the propagation direction of the spin-wave. Because of the slight phase front tilt discernible in Fig. 7b, the wave packet trajectory is tilted by 9.5° with respect to the *x* axis (compare also Fig. 6b). The result in Fig. 8 includes a fit according to equation (3) and emphasizes the evolution of the wave packet parameters. For the BV modes we obtain a carrier frequency of *f* ≈ 3.5 GHz in agreement with the spin-wave dispersion relation in Fig. 2a. Analogous to the DE mode the wave packet duration *τ* increases as the spin-wave emerges and propagates and the center of the wave packet envelope 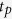 shifts to later moments in time for an increased distance from the source region. The limited scan range showing only a fraction of the wave packet envelope with few oscillations, however, restrains a quantitative analysis of the group velocity as performed on the DE wave packets and only an upper limit of 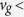 25 km/s can be extracted. Altogether, we find a propagation of the wave envelope away from the source while the extracted phase fronts from Fig. 7a clearly approach the excitation region meaning that 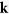 is counteraligned to 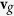. This concludes that we have directly observed backward volume modes with negative 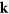-vectors that formally correspond to optical wave packets propagating in a medium with negative index of refraction. Note that the propagation of the wave packet envelope is given by the group velocity vector so that the flow of energy still points out of the source. A wave packet not emerging out of the excitation zone would violate causality and is also physically not possible in our geometry since the sample ends at *x* = 0 μm. Moreover, phase- and group velocity calculated by the dispersion relation in Fig. 2 formally exhibit a negative sign for the group velocity and a positive sign for the phase velocity. The physical meaning, however, is encoded in the oppositeness of the propagation direction and phase front motion.

### Summary

In summary, we have directly observed micron-scale Damon-Eshbach and backward volume spin-wave packets with counterpropagating phase- and group-velocities in ferromagnetic materials through a novel pulsed excitation method. The optically driven high-current pulse source provides an efficient launch of BV as well as DE spin-wave packets using intense picosecond magnetic pulses created by strong current pulses out of a metallic photocathode. The spin-wave packets are traced by stroboscopic scanning Kerr microscopy with inherently synchronized excitation- and probe-pulses generated by a femtosecond laser system. The impulsive excitation with a direct observation enables following the spin-wave creation in time and observing the build-up of higher quantized modes in the permalloy sample leading to a curvature of the phase fronts. The BV mode with directly accessible phase fronts approaching the excitation region is a promising candidate for spintronic data transport and enables model studies of negative refraction analog to optical wave propagation inside negative index metamaterials.

## Methods

### Fabrication

Rectangular permalloy (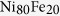) samples of 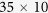 μ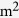 dimension and 30 nm thickness are prepared onto a 400 μm thick sapphire (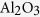) substrate (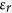 = 9.4) of 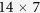 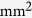 size using electron-beam lithography, physical vapor deposition (PVD), and lift-off processing. A coplanar waveguide (CPW) consisting of a 300 nm copper layer protected by a 5 nm gold cap layer is fabricated on top of the structure by ultraviolet (UV) photolithography, PVD, and lift-off processing. The 1.5 mm diameter magnesium (Mg) photocathode layer is deposited onto the source region of the waveguide by PVD through a perforated mask.

The waveguide design connects the macroscopic photocathode for pulse injection on one side with a resistive termination on the other side through a tapered section. In the latter, the waveguide conductor width is reduced down to 10 μm in order to increase the current density. The conductor is surrounded by two ground planes separated by a gap of 48.5 μm forming a CPW of 100 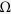 impedance and 65 GHz cut-off frequency that provides enough space for the magnetic sample inside the gap. The overlap between permalloy layer and waveguide that determines the excitation spectrum of the 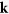-vectors measures 5 μm. Careful impedance matching across the taper is vital for minimizing losses and preserving the pulse shape. Termination of the pulses is achieved in a serial connection of two 50 Ω radio-frequency resistors with a bandwidth of 50 GHz. The 400 μm long central layout of the sample is depicted in Supplementary Fig. S1.

### Measurements

The Kerr microscope is based on a femtosecond chirped pulse amplification (CPA) ytterbium doped potassium-gadolinium tungstate (Yb:KGW) solid-state laser system that delivers pulses of 290 fs full-width-at-half-maximum (FWHM) duration at a repetition rate of 130 kHz and a wavelength of 1030 nm with a pulse energy of up to 46 μJ. By second- and fourth harmonic generation in two Beta-Barium-Borate (BBO) crystals, these pulses are converted into 515 nm green probe pulses and 258 nm UV pump pulses, respectively, that are inherently synchronized to each other since they emerge from the same source pulse. For stroboscopic measurements the pump pulse arrival time at the sample is adjustable with respect to the probe pulse using an optical delay stage that covers a time interval of 2 ns with an accuracy below one femtosecond^53^. To measure the Damon-Eshbach modes, a delay range of 1.5 ns is scanned in 5 ps steps; in case of the backward volume modes, a scan range of 1.27 ns is swept in 5 ps steps.

The probe pulses of 230 pJ pulse energy are polarization-filtered and focused onto the sample through the sapphire substrate by a microscope objective with a numerical aperture of 0.4 providing a spatial resolution of 550 nm^53^. The peak intensity in the focal spot of 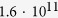 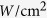 is kept right below the damage threshold of the thin sample layer. The reflected light pulse is recollected by the same objective and its polarization is analyzed by a Wollaston prism and a balanced photodetector. This enables a measurement of the out-of-plane component of the magnetization 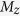 due to the polar Kerr effect. The sample can be scanned through the laser focus by an encoded two-axis piezo scanner with a travel range of 100 × 100 μm^2^ and a positioning accuracy of 25 nm. For the measurement of the Damon-Eshbach modes a window of 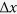 = 12 μm and 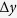 = 6 μm is scanned in 500 nm steps; in case of the backward volume modes a scan window of 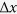 = 10 μm and 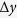 = 10 μm is swept in 500 nm steps. The data acquisition time at each scanned data point is set to 200 ms so that one SKM pixel at a given pump-probe delay is averaged over 26,000 pump-probe cycles. An external bias field is applied by a set of two 5 × 5 × 1 mm^3^ permanent magnets separated by a distance of 5 mm yielding *μ*_0_*H*_ext_ ≈ 60 mT for the DE wave packets and in a distance of 7 mm yielding *μ*_0_*H*_ext_ ≈ 20 mT for the BV modes. For BV wave packets the field is applied in −*x* direction and for DE measurements, the external field is aligned along the *y* axis. A direct *in-situ* field measurement at the sample position is not possible due to space constraints. The field amplitude is estimated from reference measurements using the same geometry of the magnets and confirmed by simulations. A comparison with the backward volume spin-wave dispersion relation in Fig. 2a suggests a smaller external field of 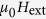 = 16 mT to fit the gyration frequency and the phase velocities in Fig. 2c. Due to the lack of a direct measurements of the local field this value seems to be more realistic.

The current pulses triggering the spin-waves are created by illuminating the Mg photocathode on the CPW conductor with UV pulses of 3 μJ pulse energy and applying an electrostatic extraction field of 6 to 7 kV/mm using a cylindrical anode in a vacuum environment with a base pressure of 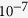 mbar. Since the photon energy of the laser pulses of 4.8 eV exceeds the work function of the Mg layer (3.66 eV), electrons are released via the photoeffect leaving a positive charge hole. This vacancy is refilled with electrons through the CPW and leads to a propagating current pulse with a bunch charge of 15 pC corresponding to an average current of 2.2 μA. This results in a quantum efficiency of the photocathode in the 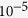 range in agreement with untreated Mg cathodes. An oxygen layer removal technique^60^ may further increase the quantum efficiency so that even stronger current pulses become available. In order to improve the detection sensitivity, the pump beam is chopped at a frequency of 1 kHz and the photodiode signal filtered by a lock-in amplifier referenced to the chopper frequency. The pulses are verified not to exceed 70 ps FWHM by a direct measurement with a 12 GHz sampling oscilloscope but simulations on the magnetic response of the permalloy layer (see Supplementary Fig. S4) suggest the actual pulse duration to be considerably shorter. The involved high frequencies are subject to the skin effect which confines the current to a thin surface layer of the conductor. The corresponding current density is estimated to exceed 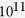 A/m^2^ at the position of the excitation region of the magnetic sample, corresponding to a local magnetic field strength of 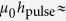 15 mT.

## Additional Information

**How to cite this article**: Wessels, P. *et al.* Direct observation of isolated Damon-Eshbach and backward volume spin-wave packets in ferromagnetic microstripes. *Sci. Rep.* **6**, 22117; doi: 10.1038/srep22117 (2016).

## Supplementary Material

Supplementary Information

Supplementary Movie S5

Supplementary Movie S6
