# Supplementary material for: Direct observation of isolated Damon-Eshbach and backward volume spin-wave packets in ferromagnetic microstripes
Source: Sci Rep. 2016 Feb 24;6:22117. doi: 10.1038/srep22117 (PMC4764955; doi:10.1038/srep22117)
Supplement: Supplementary Information [file srep22117-s1.pdf]

Direct observation of isolated Damon-Eshbach and backward volume spin-wave packets in ferromagnetic microstripes

Philipp Wessels*, Andreas Vogel, Jan-Niklas Tödt, Marek Wieland, Guido Meier, and Markus Drescher

January 11, 2016

Supplementary Information

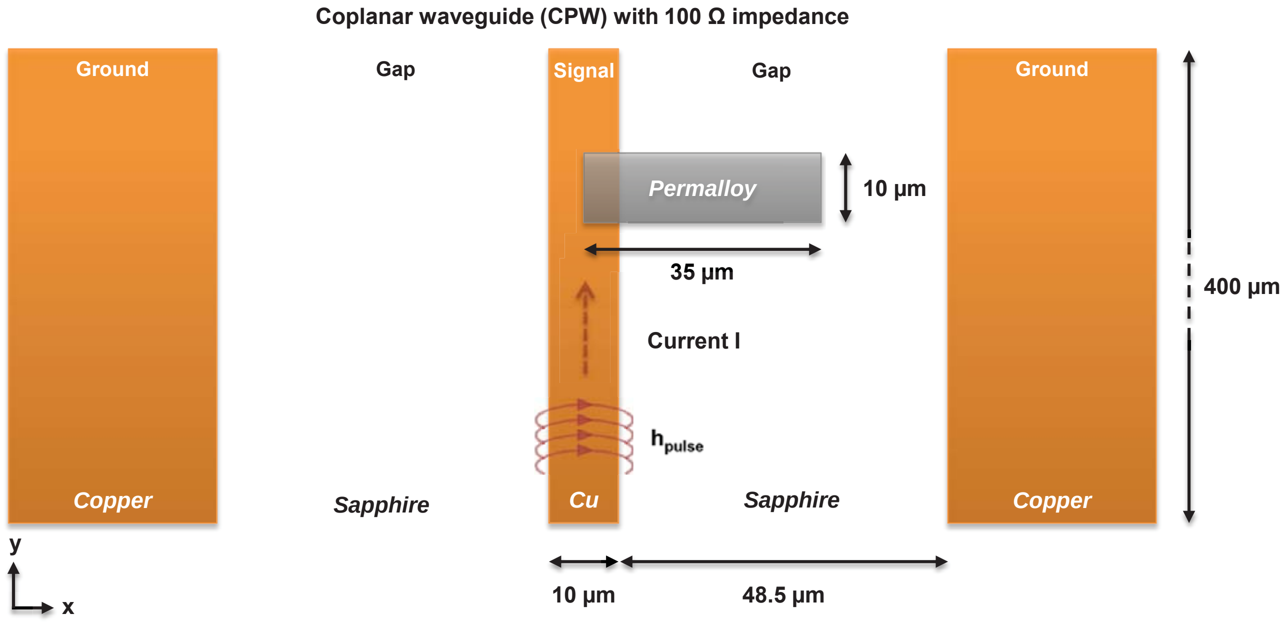

Supplementary Figure S1: **Geometry of the central part of the sample.** The sample consists of the $35 \times 10\ \mu m^2$ magnetic permalloy layer in 5 µm partial overlap with the central conductor of a coplanar waveguide (CPW). The copper waveguide is fabricated onto a sapphire substrate and features a central 10 µm wide conductor and two ground planes separated by 48.5 µm resulting in an impedance of 100 Ω.

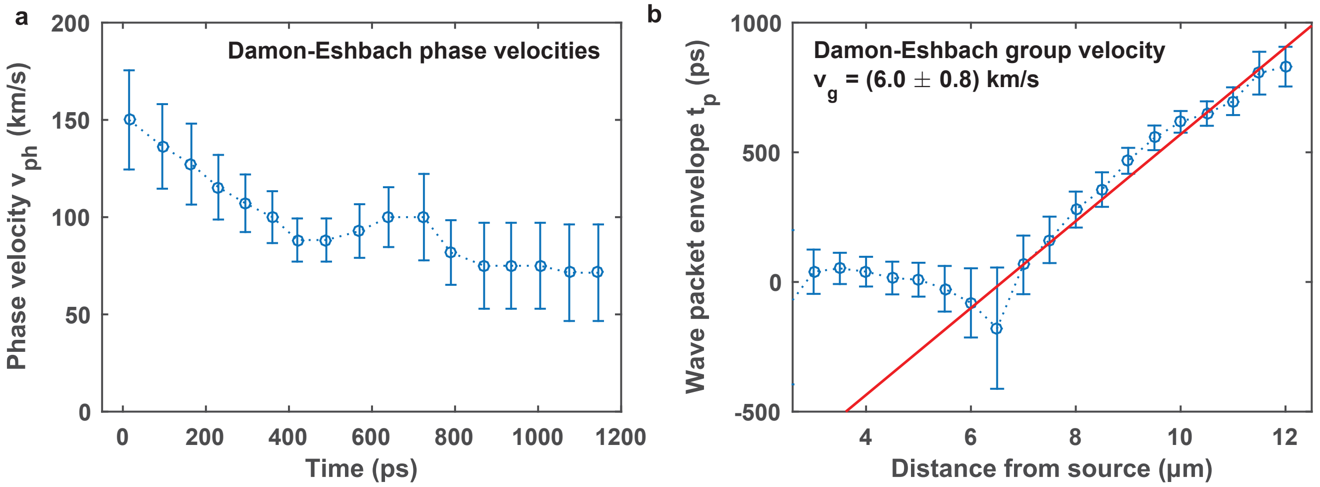

Supplementary Figure S2: **Phase and group velocities of a DE spin-wave packet.** (**a**) Phase velocities $v_{\mathrm{ph}} = \Delta x/\Delta t$ of the DE wave packet according to the dashed lines in Fig. 4a. The phase velocities are positive and range from 70 to 150 km/s. (**b**) Propagated spin-wave packet distance versus wave packet envelope time t_p (blue dots) for the DE mode. A linear fit (red solid line) according to $t_p(x) = 1/v_g \cdot x + b$ is applied to extract the group velocity of $v_g = (6.0 \pm 0.8)$ km/s.

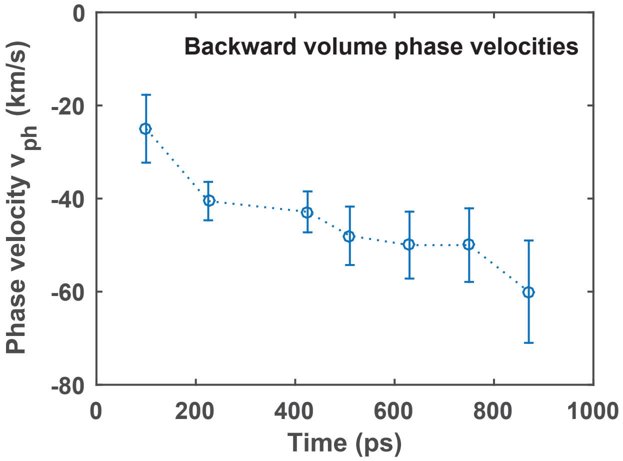

Supplementary Figure S3: **Phase velocities of the BV wave packet.** According to the dashed lines in Fig. 7a the phase velocities $v_{\mathrm{ph}} = \Delta x/\Delta t$ are negative and range from -25 to -60 km/s.

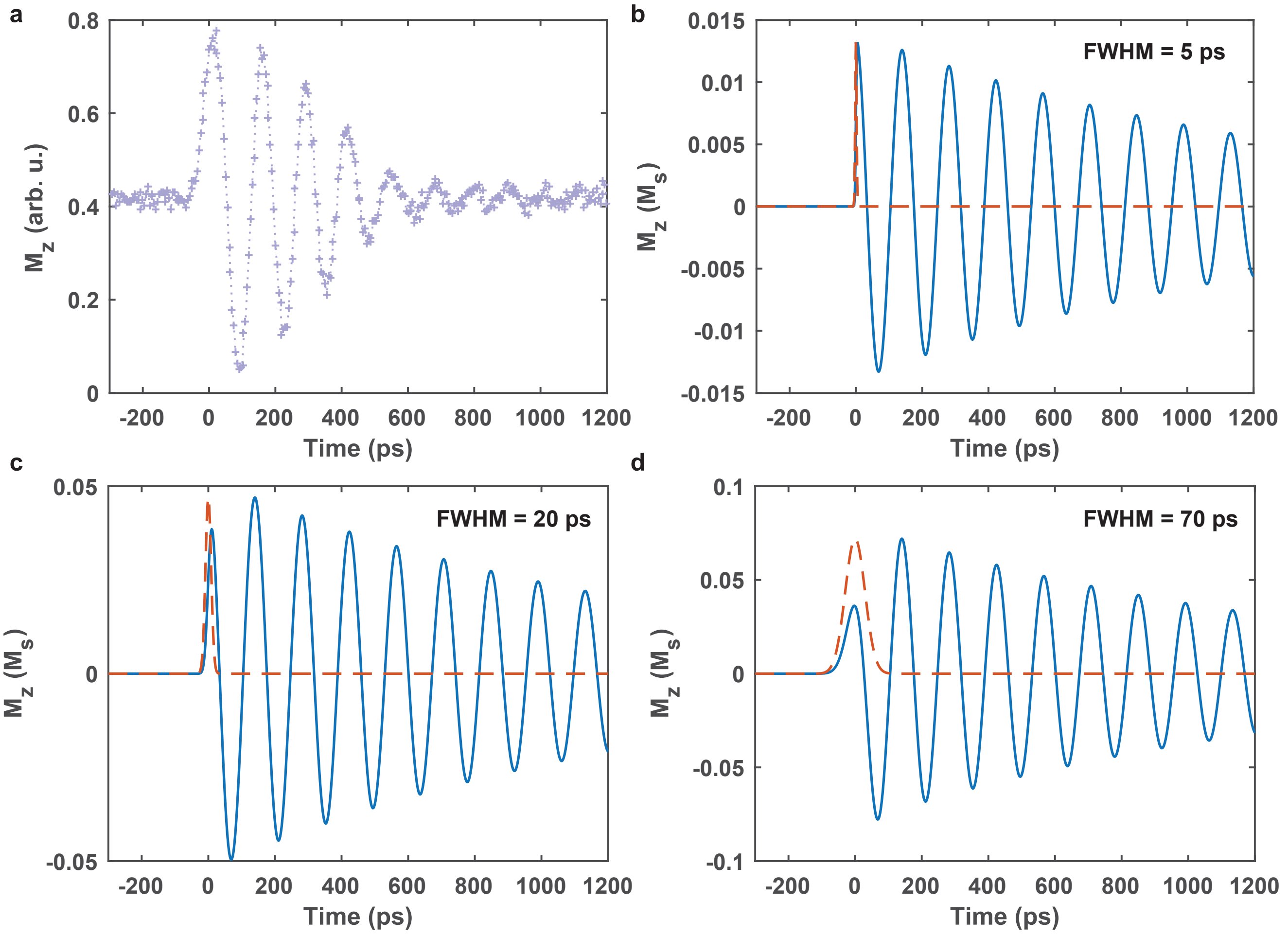

Supplementary Figure S4: **Comparison of measured and calculated permalloy response to an excitation pulse.** (**a**) Spatially averaged dynamic magnetization response $M_z(t)$ in the excitation area of Δx = 4 to 5 μm and Δy = -0.5 to 0.5 μm in DE configuration (compare Fig. 4). (**b** - **d**) Calculated magnetic precession response $M_z(t)$ (blue solid line) in units of the saturation magnetization $\mu_0 M_s$ = 1.04 T of a permalloy layer in an external bias field of $\mu_0 H_{\text{ext}} \approx$ 60 mT to a short Gaussian excitation pulse of $\mu_0 h_{\text{pulse}} \approx$ 15 mT amplitude and (**b**) 5 ps, (**c**) 20 ps and (**d**) 70 ps FWHM pulse duration (orange dashed line) obtained by numerically solving the Landau-Lifshitz-Gilbert equation. A long excitation pulse leads to undershoots of the dynamic magnetization signal due to the residual field of the pulse acting as an additional transient bias field. These features are not visible in the measured signal but observed in the literature[1,2] where longer pulses generated by an Auston switch have been used. Pulses exceeding a duration of 70 ps FWHM are excluded by a direct measurement. The higher damping in the measured signal compared to the calculations is due to the fact that the precession is transferred out of the excitation region because a spin-wave packet is released. In the calculations only the intrinsic damping of the permalloy layer (α = 0.008) is taken into account.

Supplementary Video S5: **Video showing the generation and propagation of a Damon-Eshbach spin-wave packet.** This video shows the real-space magnetization dynamics with respect to time. After arrival of the excitation pulse at the sample, the precessional motion of the magnetic moments is initiated at positions around $x = 5$ μm. In DE geometry the phase fronts in form of maxima and minima start to propagate toward higher x coordinates while after a few hundred picoseconds the whole spin-wave packet is released and the precessional motion is transferred down the x axis. This means that phase- and group velocity are parallel and point into the same direction away from the source.

Supplementary Video S6: **Video showing the generation and propagation of a backward volume spin-wave packet.** This video shows the real-space magnetization dynamics with respect to time. After arrival of the excitation pulse at the sample, the precessional motion of the magnetic moments is initiated at positions around $x = 2$ μm. In BV geometry the phase fronts in form of maxima and minima do not emerge out of the excitation region but rather approach the source from higher x coordinates. This means that phase- and group velocity are counteraligned and this unusual wave motion deduced from the dispersion relation of BV spin-wave packets becomes directly visible.

References

[1] Liu, Z., Giesen, F., Zhu, X., Sydora, R. D. & Freeman, M. R. Spin wave dynamics and the determination of intrinsic damping in locally excited permalloy thin films. *Phys. Rev. Lett.* **98**, 087201 (2007). URL http://dx.doi.org/10.1103/PhysRevLett.98.087201.

[2] Lee, K.-D. *et al.* Femtosecond pump-probe MOKE microscopy for an ultrafast spin dynamics study. *J. Korean Phys. Soc.* **49**, 2402–2407 (2006). URL http://www.kps.or.kr/jkps/abstract_view.asp?articleuid=A226818D-F262-4F8C-A784-2CD25844F084.
